# Supplementary material for: BMI Alterations and Prevalence of Overweight and Obesity Related to Service Duration at the German Armed Forces
Source: Healthcare (Basel). 2023 Jan 11;11(2):225. doi: 10.3390/healthcare11020225 (PMC9859257; doi:10.3390/healthcare11020225)
Supplement: Supplementary file 1 [file healthcare-11-00225-s001.zip › healthcare-2139673-supplementary.pdf]

healthcare

Supplementary material

Table S1. Body mass index (BMI kg/m²) at the entry to the military service for both sexes and in the years from 2010 to 2022 (Median and IQR: 25%-75%).

year	*n*	*Median*	25%	75%
2010	32,243	23.4	21.5	25.8
2011	13,030	23.5	21.5	25.8
2012	6365	23.5	21.6	25.7
2013	6010	23.4	21.6	25.7
2014	5958	23.4	21.5	25.7
2015	5325	23.5	21.5	25.8
2016	6156	23.5	21.5	25.8
2017	4418	23.2	21.3	25.7
2018	2488	23.5	21.4	25.8
2019	1523	23.4	21.3	26.1
2020	773	23.6	21.4	26.6
2021	629	23.9	21.6	26.6
2022	126	23.8	20.8	26.9
total<br>misssing	85,044<br>35	23.5	21.5	25.8
